# Knowledge, attitudes and behaviours regarding antibiotics use among Cypriot university students: a multi-disciplinary survey

**DOI:** 10.1186/s12909-022-03853-2

**Published:** 2022-12-07

**Authors:** Buket Baddal, Timo Juhani Lajunen, Mark J. M. Sullman

**Affiliations:** 1 Department of Medical Microbiology and Clinical Microbiology, Faculty of Medicine, Near East University, Nicosia, 99138 Cyprus; 2 Department of Psychology, Norwegian University of Science and Technology, Trondheim, 7012 Norway; 3 Department of Social Sciences, School of Humanities and Social Sciences, University of Nicosia, Nicosia, 2417 Cyprus

**Keywords:** Knowledge, Attitudes, Personality, Students, Antibiotics, Survey, Northern Cyprus

## Abstract

**Background:**

The present study aims to investigate the knowledge and attitudes towards antibiotics among students studying medicine, dentistry, and pharmacy at the Near East University in Northern Cyprus. The influence of personality characteristics on antibiotic use were also evaluated, in order to identify predictors of antibiotic misuse.

**Methods:**

Students were enrolled in the study during the 2020–2021 academic year. Study participants were asked to complete an online questionnaire that measured their knowledge, attitudes and practice (KAP) towards antibiotic use and antibiotic resistance. The KAP of students from the three faculties were compared using Kruskall-Wallis H statistics, Mann-Whitney U statistics, and Spearman’s rho. The influence of personality traits on the propensity to use antibiotics without a prescription, as well as their attitudes and knowledge of antibiotic use and misuse, were also investigated.

**Results:**

In total, 314 students completed the survey, 52% of which were female. The mean age of the students was 20.5 years. The results showed that among the different disciplines, medical students were significantly more knowledgeable about pharmacological agents, compared to dentistry and pharmacy students, while pharmacy students were more knowledgeable about the effectiveness of antibiotics against different pathogenic microorganisms. All student groups were aware of how antibiotic resistance develops and their role as healthcare personnel in implementing measures against resistance. Appropriate antibiotic use among the student community correlated with study year, highlighting the importance of knowledge and education in the prevention of antibiotic resistance. Personality traits were found to be a contributing factor in students’ tendency to use antibiotics without a prescription.

**Conclusion:**

This study demonstrates the importance of conveying knowledge about antimicrobials in the education programmes of future dentists, pharmacists and physicians.

**Supplementary Information:**

The online version contains supplementary material available at 10.1186/s12909-022-03853-2.

## Background

The excessive use of antibiotics in humans and animals is a global problem that has been accelerated by the COVID-19 pandemic [1, 2] and represents one of the largest threats to human health, food security and development [3]. The World Health Organization (WHO) reports that the unnecessary and inappropriate use of antibiotics increases the antibiotic resistance problem and threatens modern medicine by rendering antibiotics ineffective. With the increased selection pressure favouring the emergence of pathogenic drug-resistant bacteria, surgical site and post-operative infections are a major concern [4]. In particular, elevated levels of drug resistance in microorganisms, such as methicillin-resistant *Staphylococcus aureus*, vancomycin-resistant *Enterococcus faecium*, carbapenem-resistant *Acinetobacter* species and multi-drug resistant *Pseudomonas aeruginosa* have been found at alarmingly high levels, and have been classified as urgent and serious antimicrobial threats by the Centers for Disease Prevention and Control [5]. Increasing antimicrobial resistance to fluoroquinolones and the emergence of extended-spectrum beta-lactamase (ESBL)-producing strains among *Escherichia coli* circulating in the community are also important issues that are relevant to antimicrobial strategies [6]. The 2020 WHO annual antibacterial pipeline report revealed that the drug candidates in clinical development and those recently approved were insufficient to tackle the challenge of antimicrobial resistance, and the global shortage of innovative antibiotics was likely to further fuel the emergence and spread of drug-resistance [7].

The development of antibiotic resistance by microorganisms is a natural phenomenon that is accelerated by the frequent and inappropriate use of antibiotics [8, 9]. Therefore, interventions aimed at increasing the level of knowledge about appropriate antibiotic use among patients and healthcare professionals, as well as improving communication between the two, are important steps towards combating antibiotic resistance [10]. However, in order to generate appropriate interventions, it is essential to have a thorough understanding of both patients’ and health care professionals’ levels of knowledge, understanding, and attitudes regarding antibiotic use and antibiotic resistance [11]. Health care professionals, including nurses, midwives, clinicians and dentists, have an essential role in antimicrobial stewardship, as their daily practice can have a direct impact on antimicrobial resistance. The proper use of antibiotics is crucial for the treatment of patients, but improper use leads to increased antibiotic resistance, severe infections, complications, longer hospital stays and increased mortality rates. Furthermore, having an understanding of the education given to future healthcare professionals, in relation to these same issues, is critical for identifying major learning gaps in the education system. Substantial intercountry differences exist in antibiotic knowledge and practice. In addition, diversity in the health systems, healthcare practices and education related to the use of antibiotics means that research is required to enable a better understanding of the local context in each country or territory. In Cyprus, antibiotic resistance represents a large problem in both the community and healthcare settings. The Annual Epidemiological Report in 2018, from the European Centre for Disease Prevention and Control (ECDC), indicated that methicillin resistance among *S. aureus* isolates was between 31.2% and 43.4%, carbapenem resistance in *Acinetobacter* spp. was between 71.4% and 84.2%, while vancomycin resistance in *E. faecium* isolates was from 28.6 to 59.1%. This problem is mainly due to the high level of antibiotic consumption without prescription in the country, which ranks it among the top three countries within the European Union and the European Economic Area (EU/EEA). In Cyprus, antibiotic use without a prescription is mainly driven by over-the-counter (OTC) sales in pharmacies, and over 10% of all antibiotics consumed were not prescribed by a doctor [12]. These findings highlight the urgent need to address management and prevention strategies in Cyprus [13]. Although there has been one recent study investigating the knowledge, attitudes and behaviours relating to antibiotic use and resistance among the general public of Cyprus [14], this research did not include the 37% of the island that is predominantly inhabited by Turkish Cypriots. To date, there have been no peer-reviewed studies in Northern Cyprus that have reported the attitudes, beliefs and behaviours regarding antibiotic use among the general public, medical professionals or medical professionals in training.

In addition to differences in the educational and cultural setting in which the health system functions, there are likely to be personality differences which contribute to the prescribing behaviour of medical professionals (i.e., clinicians, pharmacists and veterinarians). The dominant framework for examining personality, as it relates to thoughts and behaviours, is the Big Five personality factors (Big Five). The Big Five consists of five broad personality constructs, which are: Openness, Conscientiousness, Extraversion, Agreeableness, and Neuroticism. There have been a large number of studies which have found these personality factors predict job performance, with Conscientiousness being the most consistent of the five [15]. Intolerance of Uncertainty is another personality construct of interest. Intolerance of Uncertainty (IU), also called Uncertainty Avoidance (UA), is an individual’s “tendency to be bothered or upset by the (as yet) unknown elements of a situation, whether the possible outcome is negative or not” [16]. In country-level studies, UA has been found to be related to excessive use of antibiotics [17–19]. It should also be noted, however, that UA was developed for country-level comparisons using national indicators, and therefore the role of UA in antibiotic use or prescription at the individual level is not known.

As cross-national differences exist in the attitudes, knowledge and use of antibiotics, the tailoring of educational interventions requires an understanding of the needs of the population in each country. This study aimed to collect information on the knowledge, attitudes and behaviours regarding the use of antibiotics, the problem of antibiotic resistance and the problem of antibiotic consumption in the student population of three healthcare programmes (medicine, pharmacy and veterinary science) in Northern Cyprus. This study also investigated the relationships personality characteristics have with the abovementioned variables and examined which factors were related to the propensity to use antibiotics without a prescription. This study will provide information that may help in the development of programmes to improve attitudes and knowledge regarding antibiotics and ultimately aid in the control of antibiotic resistance.

## Methods

### Participants and data collection

All participants of this study were Turkish Cypriots. The island of Cyprus has been divided in two since 1974, based upon ethnic identity, with the Greek Cypriots living in the southern part and the Turkish Cypriots living in the northern part of the country. The study was conducted during the 2020–2021 academic year. All participants (*n* = 314) were students at the faculties of Medicine, Pharmacy and Dentistry at the Near East University in Northern Cyprus, who were asked to complete an online questionnaire hosted by Google Forms. The students received a link to the questionnaire, which contained information on the background to the study, objectives, voluntary nature of participation, declarations of anonymity and the confidentiality of all data, and informed consent was obtained from all participants. This study was approved by the Institutional Review Board at Near East University (YDU/2020/86-1242). As the Google Forms survey link was distributed freely among all students and no personal identifiers were collected, it was not possible to estimate the response rate.

### Questionnaire

The questions used in this survey were adapted from earlier studies [16, 20, 21] and adjusted to suit our study population. The questionnaire consisted of five different sections. The first section asked participants to report their demographic details, including sex, age, educational status and nationality. In section 2, participants were asked whether they had taken antibiotics themselves in the last 12 months and if they had, whether they had taken one course or more (‘Once’, ‘2–5 times’, ‘More than 5 times’). The questionnaire can be found in Additional file 1.

Section 3 consisted of seven blocks of questions. In the first subsection, participants were presented with nine types of drugs, drawn from a range of antibacterial (amoxicillin, penicillin, tetracycline), antiviral (favipravir, remdesivir), antifungal (fluconazole), anti-parasitic (hydroxychloroquine) and anti-inflammatory (aspirin, ibuprofen) drugs. The participants were asked whether each was an antibiotic and to record their answer using one of the three options (‘Yes’, ‘No’, ‘I Don’t Know’). A recognition score (incorrect answers were coded as 0, not knowing as 1, and a correct answer as 2) was calculated and used as the dependent variable in a one-way ANCOVA analysis, in which the student group (dentistry, medicine, and pharmacy) was used as the independent variable and the number of years studied was a covariate. Subsections 2–5 measured the “accessibility of antibiotics”, “antibiotics use” and its “side effects”, which were adapted from previous research among the general public in Sweden [20]. All three scales presented participants with a number of statements and asked them to report the degree to which they agreed with each using a five-point Likert scale (‘Strongly agree’ to ‘Strongly Disagree’). The accessibility of antibiotics section presented participants with six statements, the antibiotics use section contained 14 statements (alpha reliability = 0.74) and the side effects scale contained 10 (alpha reliability = 0.80). The sixth block of questions presented seven short illness descriptions and asked participants how likely they would be to prescribe antibiotics, which were reported on a five-point Likert scale (‘Not at all likely’ to ‘Extremely Likely’) (alpha reliability = 0.94). The last question in this section asked participants to report how easy it was to obtain antibiotics without a prescription, which was answered on a five-point Likert scale (‘Extremely easy’ to ‘Extremely difficult’). Participants were also asked to indicate the ease at which they could obtain antibiotics online or through relatives and acquaintances. Whether a prescription was required to obtain antibiotics in Northern Cyprus was also assessed and answered on a five-point scale (range ‘strongly agree’ to ‘strongly disagree’).

Section 4 contained the 20-item short form of the International Personality Item Pool (Mini-IPIP), which measured the Big Five Personality Factors [21]. Participants were presented with 20 statements and asked to rate how true each statement was of themselves using a five-point Likert scale (’Very inaccurate’ to ’Very accurate’). The alpha reliability coefficients for Extraversion, Agreeableness, Conscientiousness, Neuroticism, and Intellect/Imagination were 0.67, 0.63, 0.49, 0.50, and 0.50, respectively. These relatively low reliability coefficients might be due to the small number of items. Nevertheless, the results related to the Big Five should be interpreted with some degree of caution.

Section 5 contained the 12-item Intolerance of Uncertainty Scale [16]. Intolerance of Uncertainty (IU) measures an individual’s “tendency to be bothered or upset by the (as yet) unknown elements of a situation, whether the possible outcome is negative or not.“ The IUS-12 consists of 12 short statements and asks participants to report how characteristic each of the statements were of them on a five-point Likert scale (‘Not at all characteristic of me’ to ‘Entirely characteristic of me’). The alpha reliability coefficient for the IUS-12 was 0.91.

### Statistical analysis

All analyses were performed using IBM SPSS Statistics for Windows, version 25 (IBM, Armonk, New York, USA). The variables based on Likert and frequency scales were analyzed using non-parametric tests. The knowledge, attitudes and practices of students from the three faculties were compared using Kruskall-Wallis H statistics, Mann-Whitney U statistics, and Chi-square tests. Spearman rho correlations were also used to examine relationships between the personality variables and the other variables of interest. A *P* value of 0.05 or less was considered to be significant.

## Results

### Population characteristics

A total of 314 students in their first three years studying dentistry (*n* = 159, 50.6%), pharmacy (*n* = 101, 32.2%) and medicine (*n* = 54, 17.2%) were included in this study. The distribution of students, according to the year of study, was 162 (51.6%) in the first year, 87 (27.7%) in the second year and 65 (20.7%) in the third year. Mean ages of the students were 20.6 (± 2.0), 20.5 (± 1.5) and 20.5 (± 3.4) for the faculties of dentistry, pharmacy and medicine, respectively. Furthermore, there was an even gender distribution of students in each study group. The sample characteristics are presented in Table 1.

**Table 1:** Sample characteristics

Characteristic	Dentistry	Medicine	Pharmacy
Number of participants (*n* = 314)	159	54	101
Mean age (standard deviation)	20.6 ± 2.0	20.5 ± 3.4	20.5 ± 1.5
Proportion of women (%)	54.7	44.4	50.5
Year of study (%)			
1	43.4	63.0	58.6
2	30.8	0	37.4
3	25.8	37.0	4.0

### Recognition of antibiotics

The recognition scores were low in all student groups, with only 7.6% obtaining 100% (i.e., they were able to correctly recognize all antibiotics and non-antibiotic drugs among the nine pharmaceuticals). A Kruskal-Wallis test indicated that there was a statistically significant difference in the recognition score between student groups (H(2) = 9.06, *p* = 0.011). Bonferroni corrected comparisons between student groups showed that medicine (median = 1.33) and dentistry (median = 1.33) students had higher recognition scores than pharmacy students (median = 1.11), with the Bonferroni corrected statistic (10.14) being statistically significant (*p* = 0.004) between pharmacy students and dentistry students. Difference between medicine students and pharmacy students was not statistically significant (*p* = 0.145), due to the smaller sample size. These results show that medicine and dentistry students had better knowledge about pharmaceuticals than pharmacy students.

The recognition rate of antibiotics among dentistry, medicine, and pharmacy students are shown in Table 2. Statistically significant differences (chi-square tests) among student groups were found in the knowledge of amoxicillin, aspirin, favipiravir, ibuprofen, penicillin, and tetracycline. Medical students correctly classified amoxicillin (64.8%), aspirin (68.5%), ibuprofen (50.0%), penicillin (64.8%) and tetracycline (53.7%) more often than the other student groups. Dentistry students identified favipiravir as a non-antibiotic (50.3%) more often than the other two groups. However, overall medical students were the most successful at correctly classifying these drugs, while pharmacy students were the least successful.

**Table 2:** Identification of pharmaceutical agents or medicines as antibiotics among dentistry (*n* = 159), medicine (*n* = 54), and pharmacy (*n* = 101) students: proportion of answers (%) and chi-square test statistics for distributions

Agent	Dentistry			Medicine			Pharmacy			Chi square
	**Yes (%)**	**No (%)**	**Don’t know (%)**	**Yes (%)**	**No (%)**	**Don’t know (%)**	**Yes (%)**	**No (%)**	**Don’t know (%)**	
Amoxicillin^a^	30.4	35.4	34.2	64.8	14.8	20.4	35.0	27.0	38.0	22.47***
Aspirin	28.3	57.2	14.5	14.8	68.5	16.7	43.0	43.0	14.0	14.43**
Favipiravir	6.9	50.3	42.8	20.4	42.6	37.0	15.8	37.6	46.5	10.78*
Fluconazole	3.8	48.4	47.8	9.3	38.9	51.9	6.0	45.0	49.0	3.28
Hydroxychloroquine	10.1	47.2	42.8	22.6	34.0	43.4	12.9	39.6	47.5	7.06
Ibuprofen	18.2	49.7	32.1	25.9	50.0	24.1	34.7	36.6	28.7	10.24*
Penicillin^a^	42.1	42.8	15.1	64.8	20.4	14.8	52.5	27.7	19.8	13.14*
Remdesivir	2.5	49.1	48.4	5.6	46.3	48.1	7.9	44.6	47.5	4.15
Tetracycline^a^	28.7	35.7	35.7	53.7	18.5	27.8	28.3	28.3	43.4	15.13**

df = 4; *p < 0.05; **p < 0.01; ***p < 0.001; ^a^Antibiotic agent (“yes” as correct answer”)

### Antibiotic use and effects

Knowledge about antibacterial agents was also measured by asking them to report their level of agreement (5-point scale) with seven statements regarding the use and effects of antibiotics (e.g., “antibiotics make you recover faster when you have a cold”). No statistically significant differences between student groups (dentistry, medicine, pharmacy) were found using a Kruskal-Wallis test (H(2) = 2.32, *p* = 0.314). Non-parametric correlations between the study year and agreement with the sum of effects statements was statistically non-significant (Spearman’s rho = 0.01, *p* = 0.848). Hence, neither the study programme (dentistry, medicine, and pharmacy) nor the number of years studied had a statistically significant effect on the knowledge about antibiotic use and effects.

The students reported the effectiveness of antibiotics against the different types of pathogenic microorganisms (e.g., all bacteria in the body, certain bacteria, fungi, parasites, viruses) using five statements (on a 5-point scale) (Table 3). A Kruskal-Wallis test indicated that there was a statistically significant difference in the knowledge about effectiveness between student groups (H(2) = 18.84, *p* = 0.001). Bonferroni corrected comparisons between student groups showed that medicine (median = 3.80) and dentistry (median = 3.40) students had higher knowledge scores than pharmacy students (median = 3.20). The Bonferroni corrected statistic (12.69) was statistically significant (*p* = 0.001) between medicine students and pharmacy students and between dentistry students and pharmacy students (6.44, *p* = 0.034). The medians between medicine and dentistry students did not differ statistically significantly (*p* = 0.143), after applying Bonferroni corrections. The non-parametric correlation between study years and knowledge about the effectiveness of antibiotics against different microorganisms was statistically non-significant (Spearman’s rho = 0.06, *p* = 0.307). Hence, only the study group, but not the study year, made a difference in knowledge about the effectiveness of antibiotics.

**Table 3:** Agreement with the statements about the effectiveness of antibiotics (median values), Kruskall-Wallis test (H) for median differences and the Mann-Whitney test (U) for group differences

Variable	Dentistry (D)	Medicine (M)	Pharmacy (P)	H(2)	Group Differences (U)
Antibiotics are supposed to kill all bacteria in the body	3.00	2.00	3.00	9.54**	M > P** D > P*
Antibiotics are effective against viruses	2.00	1.00	3.00	14.46***	M > P** D > P**
Antibiotics are effective against bacteria	4.00	4.00	4.00	4.47	
Antibiotics are effective against fungi	3.00	3.00	3.00	14.36***	M > P*** M > D**
Antibiotics are effective against parasites	3.00	3.00	3.00	7.93*	M > P* D > P*

Answer alternatives: 1 = strongly disagree to 5 = strongly agree; *p < 0.05; **p < 0.01; ***p < 0.001

### Resistance and the prevention measures

Participants were asked questions about the development of antibiotic resistance (four questions, e.g., “bacterial resistance can spread from animals to humans”) and the effectiveness of healthcare personnel’s measures against resistance (five questions, i.e., “reporting antibiotic resistant infections to surveillance teams”) using a 5-point Likert scale.

A Kruskal-Wallis test found no statistically significant differences between student groups (dentistry, medicine, pharmacy) for knowledge about resistance, H(2) = 4.26, *p* = 0.119. The non-parametric correlation between study year and knowledge about resistance was also statistically non-significant (Spearman’s rho = 0.09, *p* = 0.123). Hence, neither the study programme (dentistry, medicine, and pharmacy) nor the number of years studied had a statistically significant effect on the statement-based knowledge about antibiotic resistance.

Student groups (dentistry, medicine, pharmacy) did not differ from each other in terms of their knowledge about the effectiveness of measures against antimicrobial resistance (Kruskal-Wallis H(2) = 0.60, *p* = 0.739). Study years had a non-significant correlation (Spearman’s rho = 0.10, *p* = 0.076) with knowledge about resistance.

### Use of antibiotics in treatment

In addition to knowledge, participants were presented with seven different sets of symptoms (Table 4) and asked if they would use antibiotics to treat the conditions described without consulting a physician. Table 4 shows that the medium for using antibiotics without prescription. A Kruskall-Wallis test showed no statistically significant differences between dentistry, medicine, and pharmacy students in the median scores across the seven scenarios (H(2) = 1.23, *p* = 0.541). The Spearman’s rho correlation coeffect between study years and use of antibiotics was statistically significant (rho = -0.14, *p* = 0.016), indicating that the more advanced the students were in their studies, the less likely they were to use antibiotics in the given conditions without consulting a physician.

**Table 4:** Use of antibiotics in different conditions among dentistry, medicine, and pharmacy students: median values and Kruskall-Wallis test statistics (H) for median differences

Symptoms	Dentistry	Medicine	Pharmacy	H(2)
1. Sore throat, runny nose, sneezing symptoms.	2.00	2.00	2.00	1.82
2. Persistent urge to urinate, a burning sensation during urinating, foul smell, cloudy urine	2.00	1.00	2.00	2.49
3. Dental abscess, an intense pain spreading	2.00	1.00	2.00	2.09
4. Swollen joint in your right arm after an intense workout	2.00	1.50	2.00	0.12
5. Vomiting, watery diarrhoea, abdominal cramps, nausea for the last 24 h	2.00	2.00	2.00	0.23
6. Yellow/brown discolouration, thickening and crumbling at the edges of your toenail	2.00	2.00	2.00	0.48
7. Sores or rashes around the genital area, unusual discharge, pain in the lower abdomen	2.00	1.00	2.00	0.91

No statistical significant differences were found among groups

### What predicts antibiotic use?

The results described above revealed clear group differences, in terms of knowledge of antibiotics, but not in knowledge of resistance or the likelihood to use antibiotics without consulting a physician. As well as inter-group differences, differences between individuals are very likely. Therefore, we investigated which factors were related to a higher likelihood of using antibiotics without a prescription. The dependent variable (DV) was the sum of the answers to the seven scenarios (Table 4), labelled “likelihood of using antibiotics without a prescription”.

#### Correlation analyses

Pearson correlation coefficients were calculated between the likelihood of using antibiotics without a prescription and the following variables: sex (*r* = 0.09, *p* = 0.103), age (*r* = 0.01, *p* = 0.803), years in each programme (*r *= -0.14, *p* = 0.016), antibiotic use in the last 12 months (*r* = -0.01, *p* = 0.877), evaluated ease to access antibiotics (*r* = 0.01, *p* = 0.938), positive attitude to accessibility (*r* = 0.38, *p* < 0.001), recognition of antibiotics (*r* = -0.18, *p* = 0.002), knowledge about the correct use and the effectiveness of antibiotics (*r* = -0.24, *p* < 0.001), a positive attitude to antibiotics use and its effects (*r* = 0.17, *p* = 0.004), knowledge of resistance (*r* = -0.06, *p* = 0.321), knowledge about the prevention of resistance (*r* = -0.41, *p* < 0.001), IU (*r* = -0.10, *p* = 0.094), Big Five Extraversion (*r* = 0.14, *p* = 0.014), Big Five Agreeableness (*r* = -0.15, *p* = 0.007), Big Five Consciousness (*r* = -0.15, *p* = 0.010), Big Five Neuroticism (*r* = 0.04, *p* = 0.456), Big Five Openness to Experience (*r* = -0.30, *p* < 0.001).

The correlation results showed that sex, age, and a history of antibiotic use in the last 12 months had no statistically significant relationship to antibiotic use without a prescription, whereas study year had a weak negative correlation. Since the recognition of antibiotics correlated significantly with the study year, a negative correlation between the study year and the likelihood to use antibiotics without a prescription could be interpreted as an education effect.

Knowledge level, measured both as the recognition of antibiotics and knowledge regarding their correct use, and the effectiveness of antibiotics correlated negatively with the propensity to use antibiotics without a prescription. Knowledge about the prevention of resistance had the highest (negative) correlation with the likelihood to use antibiotics without a prescription. These findings underline the importance of knowledge about their correct use and antibiotic resistance: the more knowledgeable the future healthcare worker was, the less likely that they would use antibiotics without a prescription.

Among the Big Five personality variables, Extraversion correlated positively with the use of antibiotics without a prescription, while Agreeableness, Conscientiousness and Openness to Experience were negatively related to the use of antibiotics without a prescription. Extraversion is related to an underestimation of risks and a willingness to take chances, so the relationship between Extraversion and more liberal antibiotic use appears logical. Similarly, Agreeableness and Conscientiousness having negative relationships with using antibiotics without a prescription could also be expected. A person scoring highly in Conscientiousness is responsible and reliable in their work, whereas a person characterized by Agreeableness is likable and tries to find a harmonious solution to a problem (e.g., illness). Since a Conscientious person would know and follow recommendations and an Agreeable person would avoid breaking norms, it is unlikely that individuals high in these qualities would use antibiotics without a prescription, since this would violate both the norms of the profession and conflict with the instructions of the medical authority. The negative correlation between Openness to Experience and antibiotic use is more difficult to explain. Openness to Experience refers also to greater creativity, flexible thinking, and unconventionality, which might mean that people scoring high in this personality trait are more likely to question the traditional ways of treating illness, such as (mis)beliefs about the effectiveness of antibiotics.

## Discussion

The knowledge, attitudes, and practice regarding antibiotics and antibiotic resistance differs widely by country/territory, culture, socioeconomic status, education, training year, age and personality. The current study was the first to investigate the knowledge, understanding and attitudes towards antibiotic use and antibiotic resistance among university students from different health science disciplines in Northern Cyprus. The main aim of this study was to identify factors associated with the misuse of antimicrobials in order to recommend appropriate interventions to combat antibiotic resistance.

Students’ overall knowledge of antibiotics, particularly their ability to recognize different antibiotic agents among other drug types, was low in all three disciplines (medicine, dentistry and pharmacy). However, medical students were the most knowledgeable and successful at identifying commonly used pharmacological agents, while pharmacy students had the lowest scores among the three groups. With regards to the knowledge of antibiotic effectiveness against different types of microorganisms, pharmacy students had higher scores than medical students and were more successful at correctly answering questions related to the susceptibility of non-bacterial pathogens (i.e., viruses, fungi and parasites) to antibiotic agents. In a prior study, in which knowledge and attitudes towards antibiotic use were studied in undergraduate students in medical and non-medical disciplines, both groups were found to have low levels of knowledge regarding the effectiveness of antibiotics against viruses and colds/coughs [22]. Furthermore, a study conducted among United Arab Emirates medical students revealed that study year was a strong determinant of KAP regarding antibiotic use and knowledge, as final year students scored more highly than first-year students [23]. Furthermore, a study of Spanish medical students found that knowledge of antibiotic stewardship increased significantly after they received infectious diseases (ID) training in their fifth and sixth-years, in comparison to students in their first three years of education without ID training [24]. Consistent with our study, a survey of 291 Polish medical students, in their first to third years, found they had a high level of knowledge (91.1%) regarding the efficacy of antibiotics against viral infections [25]. Similarly, a KAP survey about antibiotic usage and resistance among second year medical students in India revealed high knowledge scores (63–100%) and yet 67% of the participants reported self-medicating [26]. Kamoto et al. also found high knowledge scores regarding antimicrobial use and resistance among final year medical students, but there were also substantial knowledge gaps which needed to be addressed [27]. In a study investigating knowledge about antibiotics and antimicrobial resistance among pharmacy students studying at Sri Lankan universities, students in their first two years had poor knowledge about when to use antibiotics, with more than half (57%) incorrectly indicating antibiotic use for the management of cold and flu symptoms [28].

In our study, junior students in all three health science disciplines were generally unlikely to use antibiotics without prescription, which may also show that they themselves will not unnecessarily prescribe antibiotics in the future. The mean scores were similar among medical, dentistry and pharmacy students. In contrast to our findings, a study investigating the knowledge and attitudes of senior dental students towards antibiotic prescribing guidelines found that they were inclined to prescribe antibiotics inappropriately for: periapical abscess (65.7%), dry socket (37.1%), and pulpitis (25.6%), all of which are routinely managed with operative measures alone [29]. Furthermore, a recent study by Holz et al. found that an unexpectedly high 80.2% of dental students were not familiar with the term antimicrobial stewardship [30]. Similarly, a number of studies from several different countries have also recommended improvements in the knowledge and use of antibiotics among dental students [31–33]. In terms of medical students, Haque et al. reported better knowledge scores among medical students in years 4–5, than among those in year three [34].

Antibiotic resistance is recognized as an important global public-health threat. Therefore, future healthcare professionals are expected to have a thorough understanding of the factors related to the emergence of antibiotic resistance and their role in avoiding misuse of antibiotics, in order to help prevent and control the spread of antibiotic resistance. A key finding in our study was that the students in all groups had a high level of knowledge about resistance strategies and were aware of their role as a healthcare workers to implement measures against resistance. In a 2019 study, Gupta et al. concluded that the majority of medical students, from first to the last year of study, were knowledgeable about the mechanisms of antibiotic resistance and were also aware that the misuse of antibiotics can lead to antibiotic resistance to a specific pathogen [35]. In contrast, a study by Higuita-Gutierrez et al. found that a large proportion of medical students in Colombia believed that the university training they received was insufficient, regarding antibiotic use and bacterial resistance [36]. Moreover, Sakeena et al. found that junior (first and second year) pharmacy students had significant gaps in their understanding of antimicrobial resistance, as 75% of them failed to recognize antibiotic resistance as an issue in their country. However, their study also showed that their level of knowledge increased as the years of education progressed [28]. Similar results were observed by Kandasamy et al., in which 54% of pharmacy students failed to recognize antibiotic resistance as a national problem [37]. In a separate study involving students studying pharmacy, veterinary science and biology, researchers found that most students were familiar with the term AMR and 79.4% were able to identify a poorly designed dosing regimen as a contributing factor towards AMR [38]. This finding contrasts with surveys performed in other programmes (e.g., biology or mathematics), in which students reported that the body develops resistance to antibiotics and that antibiotic therapy can be stopped prior to the end of treatment, if they felt better [39]. In another study, students in the early stages of the training cycle obtained low scores in all knowledge, attitude and practice indexes regarding antibiotic use and antibiotic resistance, which indicates that the training cycle is an important factor associated with the KAP of university students [40]. This is also consistent with the findings of another study which found that Chinese medical students’ knowledge improved as they progressed in their studies, with the highest scores obtained in the third and fourth years [41]. However, final year medical and pharmacy students in the East African region were found to have little knowledge about antibiotic resistance and their use in clinical settings, which revealed gaps in their practical training [42].

Another key finding of our study was the negative correlation that the recognition and knowledge of antibiotics had, in terms of their correct use and effectiveness, with the likelihood of using antibiotics without a prescription. Similarly, students’ knowledge about the prevention of antibiotic resistance had the highest negative correlation with correct antibiotic use. A negative correlation between the study year and the likelihood of using antibiotics without prescription was also observed. These results highlight the importance of knowledge about correct antibiotic use and antibiotic resistance among the student population. The more knowledgeable the student was, the less likely they were to use antibiotics without a prescription. Furthermore, a study undertaken among non-medical (NM) university students in Serbia reported numerous misconceptions among the student population. The authors even advocated the development of elective courses in order to help improve non-medical students’ knowledge about correct antibiotic use and the need to avoid their misuse [43].

Personality traits have previously been associated with the adherence to antibiotic therapy [44]. In our study, the relationship that the Big Five personality variables have with the tendency to use antibiotics without a prescription was also investigated. In all student groups, Extraversion, which is related to the underestimation of risks, was positively correlated with the use of antibiotics without a prescription. In addition, Conscientiousness and Agreeableness, which both indicate responsibility and reliability in their work as well as the avoidance of conflicting with occupational norms, were negatively correlated with the use of antibiotics without a prescription. Also, Openness to Experience (being unconventional, creative and intellectually oriented) correlated negatively with the use of antibiotics without a prescription. These results also indicate that personality traits can have a substantial effect on the tendency of university students, who will be the future healthcare workers, to use antibiotics without a prescription.

Literature on the consumption of antibiotics in Northern and Southern Cyprus is limited. Recently published data showed that 47.9% of the general population of Northern Cyprus have used antibiotics in the last 6 months [45], whereas this rate was lower (32%) in the southern part of the island [14]. The 2017 ECDC European Surveillance of Antimicrobial Consumption Network (ESAC-Net) Surveillance Report revealed that Cyprus had the second highest community consumption of antibiotics in the EU/EAA region with 33 defined daily doses (DDDs) per 1000 inhabitants per day, which has increased in recent years [46]. The lack of a national reference laboratory and centralized reporting system in Cyprus also renders the tracking of antibiotic resistant microorganisms difficult. Published data suggests that the antimicrobial resistance patterns across the two parts of the island differ. In Gram negative bacteria, antibiotic resistance rates for *P. aeruginosa* isolates during the period 2016–2019 were higher for Northern Cyprus than in Southern Cyprus (piperacillin + tazobactam 17.25%, 19.7%; ceftazidime 14.7%, 25.6%; carbapenem 17.6%, 25.5%; aminoglycoside 4.2%, 13.5; fluoroquinolone 10.8%, 25.6%; Northern Cyprus and Southern Cyprus, respectively) [47, 48]. Similar patterns were observed for *A. baumanii* complex carbapenem resistance for the northern (70.7%) and southern (77.7%) parts of the island [49]. As OTC use of antibiotics is a major driver of high antimicrobial consumption, and consequently the high antimicrobial resistance observed in Cyprus, actions to combat the non-prudent use of antibiotics should be taken. Approaches such as implementing educational programmes for healthcare professionals, the use of electronic medical records to monitor treatments more effectively and enforcing the law on OTC sales should be followed.

This study has some limitations that should be taken into account while interpreting the findings. One of the main limitations is that we only included students from the health sciences disciplines at a single private university in Northern Cyprus. The inclusion of students from other programmes within the university, as well as from different universities across the island, would have provided a more accurate picture of the KAP among the local student community. Secondly, the sample consisted of first, second and third year students, whose programmes were mostly composed of preclinical studies. Therefore, these students may not have studied the clinical use of antibiotics. A broader sample, including students from more advanced classes, might have demonstrated the effect of education more clearly than the present sample. Nevertheless, the survey questions were mostly extracted from previous studies conducted among the general public, most of whom did not have any health-related education. Therefore, it is safe to assume that even first-year dentistry, medicine and pharmacy students are more able to answer questions about the use of antibiotics and the related risks than members of the general public. The present study demonstrates the importance of conveying knowledge about antimicrobials in the education programmes for future dentists, pharmacists and physicians.

Due to the nature of online surveys, which are self-administered, the accuracy of the results are heavily dependent upon the honesty of the respondents. The effect of this limitation has been minimized by ensuring the anonymity of the participants, since no personal identifiers were collected. Recall bias on the use of antibiotics may also result in over- or underestimating the frequency of antibiotic use.

This study also has several strengths, including the fact that this is the first study to investigate and compare the knowledge and attitudes of students regarding antibiotics and antibiotic resistance in health science programmes in Northern Cyprus. Therefore, the current study provides important information regarding a target population which has not previously been investigated. This study was performed during the early stages of the three study programmes, and hence mostly measured the KAP of students prior to specialty training. Finally, this study is also one of a very small number to have investigated the relationship that aspects of personality have with antibiotic use and misuse. This topic appears to be a potentially fruitful area for future research.

## Conclusion

Our survey has shown that medical students were the most knowledgeable about pharmacological agents and pharmacy students were the most knowledgeable about the effectiveness of antibiotics against different pathogenic microorganisms. All student groups were equally aware of how antibiotic resistance develops and their role as healthcare personnel to implement measures to help prevent resistance. Appropriate antibiotic use among the student community correlated with study year, highlighting the importance of education in the prevention of antibiotic resistance. Personality traits were found to be associated with the tendency to use antibiotics without a prescription.

## Supplementary Information

**Additional file 1.** Questionnaire.

## Data Availability

The datasets generated and analyzed during the current study are not publicly available due to the fact that the datasets include multiple extensive files with a large number of variables, but are available from the corresponding author upon reasonable request.
