# Supplementary material for: Knowledge, attitudes and behaviours regarding antibiotics use among Cypriot university students: a multi-disciplinary survey
Source: BMC Med Educ. 2022 Dec 7;22:847. doi: 10.1186/s12909-022-03853-2 (PMC9730643; doi:10.1186/s12909-022-03853-2)
Supplement: Supplementary file 1 — Additional file 1. Questionnaire. [file 12909_2022_3853_MOESM1_ESM.pdf]

Questionnaire

1. Age (in years): ____________________
2. Sex: □ Male □ Female □ Other □ Prefer not to say
3. What are you studying?

   Dentistry
   Medicine
   Pharmacy
   Other (please specify)________________________________

4. What year are you in your studies? (1st year, 2nd year, 3rd year, 4th year, 5th year)
5. What is your nationality? ___________________________________
6. What is your native language?________________________________
7. Have you taken antibiotics in the last 12 months? Please tick one option.

   □ Yes
   □ No □ Proceed to 7
   □ Don’t know □ Proceed to 8

8. How many times have you consumed antibiotics during the past 12 months? Please tick only one option.

   □ Never
   □ Once
   □ 2-5 times
   □ More than 5 times

9. The following drugs are antibiotics

Scale □ Yes, □ No □ Don't know

1. Tetracycline
2. Penicillin
3. Ibuprofen
4. Fluconazole
5. Aspirin
6. Remdesivir
7. Amoxicillin
8. Ibuprofen
9. Favipiravir
10. Hydroxychloroquine

10. Antibiotic accessibility (1=Strongly agree to 5=Strongly disagree)

a. Leftover antibiotics can be saved for personal use in the future or given to someone else.
b. Leftover antibiotics should be taken back to the pharmacy.
c. I is good that one needs a prescription to acquire antibiotics from pharmacies in Cyprus.
d. Itis good to be able to buy antibiotics online, without having to see a doctor.
e. It is good to be able to acquire antibiotics from relatives or acquaintances, without having to be examined by a doctor.
f. It is good that one can buy antibiotics without a prescription in pharmacies in Northern Cyprus.

11. Antibiotic use and its effects (1=Strongly agree to 5=Strongly disagree)

a. Antibiotics make you recover faster when having a cold.
b. If your mucus becomes coloured when having a cold, one always needs antibiotics to get rid of the cold.

c. The body can usually fight mild infections on its own without antibiotics.
d. A persistent cough (longer than one week) always needs to be treated with antibiotics to disappear.
e. It is appropriate to take antibiotics when having a sore throat and a cold, otherwise one may suffer complications.
f. It is appropriate to take antibiotics for tonsillitis, otherwise one may suffer complications.
g. An ear infection in a 3-6 year old child always needs to be treated with antibiotics.
h. Cystitis/lower urinary tract infections in women can heal themselves without antibiotics.
i. By taking antibiotics one can often avoid staying home from work.
j. Antibiotics are supposed to kill all bacteria in the body.
k. Antibiotics are effective against viruses
l. Antibiotics are effective against bacteria
m. Antibiotics are effective against fungi.
n. Antibiotics are effective against parasites.

12. Side effects and resistance (1=Strongly agree to 5=Strongly disagree)
a. Antibiotics often cause side effects, such as diarrhoea.
b. Antibiotics cause negative effects on the body's own bacterial flora.
c. If one feels better after only partially completing an antibiotic course, one can terminate the therapy immediately.
d. Bacteria can become resistant to antibiotics.
e. The more antibiotics we use in society, the higher the risk that resistance develops and spreads.
f. People can become resistant to antibiotics.
g. The frequent use of antibiotics in animals (chicken, cows, pigs) can make antibiotics less effective in humans
h. Bacterial resistance can spread from animals to humans.
i. Bacterial resistance can spread from person to person.
j. People travelling outside Cyprus risk bringing resistance to Cyprus.

13. Prevention and Reduction of antibiotic resistance.

Health care professionals can prevent and control the spread of antibiotic resistance by:

a. By ensuring their hands, instruments, and environment are cleaned/disinfected/sterilized.
b. Only prescribing and dispensing antibiotics when they are needed, according to current guidelines.
c. Reporting antibiotic-resistant infections to surveillance teams.
d. Talking to their patients about how to take antibiotics correctly, antibiotic resistance and the dangers of misuse.
e. Talking to their patients about preventing infections (for example, vaccination, hand washing, safer sex, and covering nose and mouth when sneezing).

14. How likely is it that you would use antibiotics in the following conditions? (Scale - 1=not at all likely 5=very likely)

a. You have a sore throat, runny nose and sneezing symptoms. Would you use an antibiotic without consulting a doctor?

b. You have persistent urge to urinate and a burning sensation during urinating. Urine appears cloudy and has a foul smell. Would you use an antibiotic without consulting a doctor?

c. You have a dental abscess and an intense throbbing pain which is spreading to your ear, jaw and neck area. Would you use an antibiotic without consulting a doctor?

d. You have a swollen joint in your right arm after an intense workout. Would you use an antibiotic without consulting a doctor?

e. You have vomiting, watery diarrhoea with abdominal cramps and nausea for the last 24 hours. Would you use an antibiotic without consulting a doctor?

f. You have yellow/brown discolouration, thickening and crumbling at the edges of your toe nail. Would you use an antibiotic without consulting a doctor?

g. You have sores or rashes around the genital area with an unusual discharge and pain in the lower abdomen. Would you use an antibiotic without consulting a doctor?

How easy is it to access antibiotics [without a prescription from a doctor] in Northern Cyprus? (scale? Extremely easy to Extremely difficult)

15. Please read the following statements and indicate how accurately they describe your personality

Scale 1=Very inaccurate, 2=Moderately inaccurate, 3=Neither inaccurate nor accurate, 4=Moderately accurate, 5=Very accurate.

a. I am the life of the party
b. I talk to a lot of different people at parties
c. I don't talk a lot
d. I keep in the background
e. I sympathize with others' feelings
f. I feel others' emotions
g. I am not really interested in others
h. I am not interested in other people's problems
i. I get chores done right away
j. I like order
k. I often forget to put things back in their proper place
l. I make a mess of things
m. I have frequent mood swings
n. I get upset easily
o. I am relaxed most of the time
p. I seldom feel blue
q. I have a vivid imagination
r. I have difficulty understanding abstract ideas

s. I am not interested in abstract ideas

t. I do not have a good imagination

Please circle the number that best corresponds to how much you agree with each item.

	Not at all characteristic of me	A little characteristic of me	Somewhat characteristic of me	Very characteristic of me	Entirely characteristic of me
1. Unforeseen events upset me greatly.	1	2	3	4	5
2. It frustrates me not having all the information I need.	1	2	3	4	5
3. Uncertainty keeps me from living a full life.	1	2	3	4	5
4. One should always look ahead so as to avoid surprises.	1	2	3	4	5
5. A small unforeseen event can spoil everything, even with the best of planning.	1	2	3	4	5
6. When it's time to act, uncertainty paralyses me.	1	2	3	4	5
7. When I am uncertain I can't function very well.	1	2	3	4	5
8. I always want to know what the future has in store for me.	1	2	3	4	5
9. I can't stand being taken by surprise.	1	2	3	4	5
10. The smallest doubt can stop me from acting.	1	2	3	4	5
11. I should be able to organize everything in advance.	1	2	3	4	5
12. I must get away from all uncertain situations.	1	2	3	4	5
